# A multi-disciplinary program for opioid sparse arthroplasty results in reduced long-term opioid consumption: a four year prospective study

**DOI:** 10.1186/s12871-023-02062-8

**Published:** 2023-03-29

**Authors:** D-Yin Lin, Anthony J. Samson, Freeda D’Mello, Brigid Brown, Matthew G. Cehic, Christopher Wilson, Hidde M. Kroon, Ruurd L. Jaarsma

**Affiliations:** 1 Department of Anaesthesia, Flinders Medical Centre, Flinders Drive Bedford Park, Adelaide, SA 5042 Australia; 2 Discipline of Perioperative Medicine, College of Medicine and Public Health, Flinders University, Adelaide, South Australia Australia; 3 Department of Orthopaedic and Trauma Surgery, Flinders Medical Centre, Adelaide, South Australia Australia; 4 Department of Surgery, Royal Adelaide Hospital, Adelaide, South Australia Australia; 5 Discipline of Surgery, Adelaide Medical School Faculty of Health and Medical Science, University of Adelaide, Adelaide, South Australia Australia

## Abstract

**Introduction:**

The current opioid epidemic poses patient safety and economic burdens to healthcare systems worldwide. Postoperative prescriptions of opioids contribute, with reported opioid prescription rates following arthroplasty as high as 89%. In this multi-centre prospective study, an opioid sparing protocol was implemented for patients undergoing knee or hip arthroplasty. The primary outcome is to report our patient outcomes in the context of this protocol, and to examine the rate of opioid prescription on discharge from our hospitals following joint arthroplasty surgery. This is possibly associated with the efficacy of the newly implemented Arthroplasty Patient Care Protocol.

**Methods:**

Over three years, patients underwent perioperative education with the expectation to be opioid-free after surgery. Intraoperative regional analgesia, early postoperative mobilisation and multimodal analgesia were mandatory. Long-term opioid medication use was monitored and PROMs (Oxford Knee/Hip Score (OKS/OHS), EQ-5D-5 L) were evaluated pre-operatively, and at 6 weeks, 6 months and 1 year postoperatively. Primary and secondary outcomes were opiate use and PROMs at different time points.

**Results:**

A total of 1,444 patients participated. Two (0.2%) knee patients used opioids to one year. Zero hip patients used opioids postoperatively at any time point after six weeks (p < 0.0001). The OKS and EQ-5D-5 L both improved for knee patients from 16 (12–22) pre-operatively to 35 (27–43) at 1 year postoperatively, and 70 (60–80) preoperatively to 80 (70–90) at 1 year postoperatively (p < 0.0001). The OHS and EQ-5D-5 L both improved for hip patients from 12 (8–19) preoperatively to 44 (36–47) at 1 year postoperatively, and 65 (50–75) preoperatively to 85 (75–90) at 1 year postoperatively (p < 0.0001). Satisfaction improved between all pre- and postoperative time points for both knee and hip patients (p < 0.0001).

**Conclusions:**

Knee and hip arthroplasty patients receiving a peri-operative education program can effectively and satisfactorily be managed without long-term opioids when coupled with multimodal perioperative management, making this a valuable approach to reduce chronic opioid use.

**Supplementary Information:**

The online version contains supplementary material available at 10.1186/s12871-023-02062-8.

## Introduction

The current opioid epidemic poses significant economic and patient safety burdens to healthcare systems. [1, 2] This is an issue of growing concern around the world. In the United States, for instance, opioid prescriptions have tripled from 1999 to 2014, leading to a three-fold increase of opioid-related overdoses, and in Australia over 70% of opioid overdoses are prescription related. [3]

Orthopedic surgery is in the top 5 specialties who provide opioid prescriptions, and one of the largest providers of postoperative narcotics. [4, 5] Arthroplasty surgery is associated with high incidences of new opioid prescriptions in previously opioid naïve patients, with reported incidences of 17.4 to as high as 89%, with an upwards trend in opioid prescription rates in some countries. [6, 7] In the last decade, there has been a conscious shift away from opioid based medications to treat postoperative pain in orthopaedic surgery. [8] In an effort to reduce rates of new opioid prescriptions, some studies have reported successful implementation of novel opioid sparing protocols for postoperative care following procedures normally accompanied with high rates of opioid prescriptions, including arthroplasty surgery. [9–12] These studies show a tendency towards higher rates of opioid prescription. Our study describes a protocol that aims to safely reduce the incidence of postoperative opioid prescription while achieving good patient satisfaction and patient related outcomes.

Sabatino et al. [4] described a median prescription rate of ninety 5-mg oxycodone or equivalent pills per patient who underwent total hip or knee arthroplasty with a large proportion of these medications going unused. This would seem to indicate that opioids are perhaps overprescribed following TKR/THR, and that there is room for reducing these prescriptions postoperatively.

In view of this growing opioid problem, the Departments of Anesthesiology and Orthopedic Surgery of our large teaching hospitals have implemented an Arthroplasty Patient Care Protocol (Addendum 1), focused on intensive pre-operative education and early postoperative mobilisation with the aim to limit postoperative opioid subscriptions and with the expectation of an opioid-free postoperative trajectory. The aim of this study was to evaluate if this protocol is indeed effective in decreasing longer-term opioid use, while maintaining patient satisfaction and early recovery.

## Outcomes

The primary outcome is to report our patient related outcomes in the context of this protocol, and to examine the rate of opioid prescription on discharge from our hospitals following joint arthroplasty surgery. Time points are preoperative, six weeks postoperative, three months postoperative and one year postoperative.

Three validated patient reported outcome measures (PROMs) scores were taken: the Oxford Knee Score (OKS) or Oxford Hip Score (OHS) [13], the EQ-5D-5 L score [14], and a 5-point Likert scale for patient satisfaction. Also recorded were the use of opioids at all time points.

## Patients and methods

This is a multi-centre prospective observational study. This study was conducted at two tertiary teaching hospitals (FMC and NH) in Adelaide, Australia. Orthopaedic surgeons and anesthesiologists work routinely across both sites, performing 500–600 arthroplasty procedures per year. Due to SARS Covid-19 related restrictions on elective operations, this number was reduced in 2020 to 307.

All consecutive adult patients who underwent elective knee or hip arthroplasty were approached for informed consent to participate over a three-year period. Patients were prospectively enrolled from 8th January 2018 to 1st of October 2020, with a 1 year follow up. The local Human Research Ethics Committee granted multi-centre approval (SALHN/329.17).

At both hospitals, the Arthroplasty Patient Care Protocol (Addendum 1) was implemented in 2018.

This protocol, based on previous publications [15–18], was created with multidisciplinary input from orthopaedic surgeons, anesthesiologists, physiotherapists and nursing staff. In short, the protocol involves the following. Preoperative: To qualify for surgery, patients are to taper or cease opioids and attend compulsory educational sessions. These sessions are in a group-based format and include explanations of prehabilitation techniques, the operative procedure including the anesthetic, early postoperative mobilisation, and postoperative pain and pain relief. There are also interactive question and answer components with the treating orthopaedic surgeon, physiotherapists and nurses. Emphasis is placed on the expectation for patients to be comfortably opioid-free long term postoperatively, but with low threshold to discuss pain management options. The use of simple analgesic alternatives (paracetamol and non-steroidal anti-inflammatory drugs if not contraindicated) is encouraged with education provided for dosing intervals. All hospital prescribers undergo mandatory education regarding the opioid sparse program prior to patient contact. Intra-operative: Spinal anesthesia without intrathecal morphine is the preferred anesthesia method with local infiltration analgesia (LIA) by the surgeon. Standard regional anesthesia was an adductor canal block for knee, and from 2019 a pericapsular nerve group (PENG) block for hip arthroplasty was added. [19, 20] Postoperative: Anesthesiologists and the Acute Pain Service review all patients daily, reinforcing the expectation of opioid-free recovery with administration of regular simple oral analgesia. Patients are allowed opioids as an inpatient if indicated. The inpatient stay is extended if indicated by acute (on chronic) pain and subsequent opioid titration, until the multidisciplinary team determines that the patient is optimised to proceed comfortably as an opioid sparse outpatient. Patients are discharged with a maximum of ten tablets of opioid medication (oxycodone or tramadol) without repeat prescriptions. There is some flexibility if inpatient opioid use indicates that 10 tablets is unlikely to be adequate, but this is by extremely infrequent exception. Postoperative: Orthopaedic surgical follow-up takes place at six weeks and three months. If a patient requires review for postoperative pain, there are appointment slots available in the outpatient clinic. If these are not possible for logistical reasons, the patient is encouraged to see their treating primary care physician who has had a letter sent to them communicating that a joint replacement has been carried out and the opioid sparse expectation of the postoperative trajectory.

This approach is also supported on a national level by a revision to the Pharmaceutical Benefit Scheme in Australia, implemented since 1 June 2020. Postsurgical patients with acute non-cancer pain only qualify for financial cover for a half-pack (10 tablets) of opioid based medication. Any prescription above this amount is paid for by the patient themselves. Long term opioid prescribing requires registration of the patient, and completion of an ‘Authority Prescription’ process with written or telephone approval from a central government agency. Currently, a program known as Script Check is being implemented in Australia, which allows a registered medical practioner to check if a patient has had multiple opioid prescriptions. It allows the program user to check in real-time the prescribing history of a particular patient for high-risk prescriptions, including all opioid based medications. It will shortly be mandatory to cross reference a patient using this system prior to providing such a prescription.

Protocol follow-up for this study took place at four different time points: preoperatively, and at six weeks, six months and one year postoperatively. Data were recorded by a dedicated research assistant, using scripted questionnaires either via telephone or via a posted written survey. The same script was used at all four time points. Three validated patient reported outcome measures (PROMs) scores were taken: the Oxford Knee Score (OKS) or Oxford Hip Score (OHS) [13], the EQ-5D-5 L score [14], a 5-point Likert scale for patient satisfaction, and the use of opioids at all time points. Data were entered into a password secured database stored on the hospital computer network. The database was subdivided into knee or hip arthroplasty. A random sample of patients (n = 52) were called by a separate research assistant to corroborate entered data.

The primary outcome was opiate use at the different time points. Secondary outcomes were the PROMs at the different time points.

The analyses were performed using SPSS version 27 (IBM Corp., Armonk, NY, USA) and GraphPad Prism version 8 (GraphPad Software, La Jolla, Calif, USA). The Shapiro-Wilk test showed that all continuous variables were nonparametric and are therefore described as median with interquartile range (IQR). Categorical variables are described as frequency with percentages. Univariate analyses were carried out using the chi-squared test or Fisher’s exact test (for n < 20) for categorical variables, and the Mann-Whitney U-test for continuous variables. A p-value of < 0.05 was considered statistically significant.

## Results

Out of 1,728 consecutive patients who were invited to participate, 1,444 (84%) provided informed consent.

### Knee arthroplasty group

A total of 917 patients underwent knee arthroplasty. The majority was female (n = 613; 67%) with a median age of 73 (IQR 65–80), median body mass index (BMI) of 32 kg/m^2^ (IQR 28–36) and most had surgery for a primary replacement, 909 (99%). Of the knee arthroplasty patients, 232 (25%) used opioids preoperatively. At baseline, 655 (72%) patients reported no or minimal anxiety/depression (EQ-5D-5 L score), moderate anxiety/depression was reported by 198 (21%) and 64 (7%) had severe or extreme preoperative anxiety/depression. (Table 1)

**Table 1:** Characteristics for knee arthroplasty patients

	Knees (n = 917)
**Age** in years, median (IQR)	73 (65–80)
**Gender, n (%)**	
Male Female	304 (33) 613 (67)
**Weight** in kg, median (IQR)	88.9 (75.1–100)
**BMI** in kg/m^2^, median (IQR)	32 (28–36)
**Type of surgery, n (%)**	
Primary Revision	909 (99) 8 (1)
**Operative side, n (%)**	
Left Right	403 (44) 514 (56)

Abbreviations: IQR: interquartile range

Of the 917 patients who consented to participate in the questionnaire follow-up, 59 (6.4%) were lost to follow-up at six weeks (a slightly higher number of 86 (9.3%) for the satisfaction component). 179 (19.5%) were lost to follow up at six months, and 353 (38.5%) did not respond to the 1 year questionnaire.

Two (0.2%) patients used long-term opioids postoperatively, at six weeks, six months and one year after surgery (p < 0.0001 compared to preoperatively). (Table 2) Both were using opioids preoperatively.

**Table 2:** Pre- and postoperative opiate use and patient Reported Outcome Measures (PROMs) for knee arthroplasty

	Knees (n = 917)	p-value (comparison to pre-operative)
**Opiate use**, n (%)^a^		
pre-operative	232 (25)	-
6 weeks postoperative^1^	2 (0.2)	< 0.0001^*^
6 months postoperative^2^	2 (0.2)	< 0.0001^*^
1 year postoperative^3^	2 (0.2)	< 0.0001^*^
**Oxford Knee Score total**, median (IQR)^b^		
pre-operative	16 (12–22)	-
6 weeks postoperative^1^	27 (21–33)	< 0.0001^*^
6 months postoperative^2^	34 (27–40)	< 0.0001^*^
1 year postoperative^3^	35 (27–43)	< 0.0001^*^
**EQ-5L-5D Health Questionnaire total**,		
median (IQR)^b^		
pre-operative	70 (60–80)	-
6 weeks postoperative^1^	80 (70–90)	< 0.0001^*^
6 months postoperative^2^	80 (70–90)	< 0.0001^*^
1 year postoperative^3^	80 (70–90)	< 0.0001^*^

The total Oxford Knee Score improved significantly after surgery from 16 points (range 12–22) preoperatively to 35 points (range 27–43) at one year (p < 0.0001). Similarly, the total EQ-5D-5 L score improved from 70 points (range 60–80) preoperatively, to 80 points (range 70–90) at one year (p < 0.0001). (Table 3)

**Table 3:** Pre- and postoperative patient Reported Outcome Measures (PROMs) for knee arthroplasty

	Pre-operatively	6 weeks	6 months	1 year	p-value
**EQ-5L-5D pain/discomfort score**, n (%)^c^	n = 917	n = 885	n = 747	n = 578	
No pain	12 (1)	60 (7)	164 (22)	186 (32)	< 0.0001^*^
Slight pain	74 (8)	373 (42)	365 (49)	248 (43)	
Moderate pain	349 (38)		166 (22)	105 (18)	
Severe pain	410 (45)	358 (41)	46 (6)	37 (6)	
Extreme pain	72 (8)		6 (1)	2 (1)	
* Lost to follow up*	-	83 (9) 11 (1) *32*	*170*	*339*	
**EQ-5L-5D anxiety/depression score**, n (%)^c^	n = 917	n = 883	n = 742	n = 574	
Not anxious/depressed	386 (42)	530 (60)	468 (63)	367 (64)	< 0.0001^*^
Slightly	269 (30)	219 (25)	171 (23)	152 (26)	
anxious/depressed	198 (21)		86 (12)	49 (9)	
Moderately	47 (5)	103 (11)	17 (2)	6 (1)	
anxious/depressed	17 (2)	26 (3)	0	0	
Severely	-	5 (1)	*175*	*343*	
anxious/depressed		*34*			
Extremely					
anxious/depressed					
* Lost to follow up*					
**Likert patient satisfaction**, n (%)^c^	n = 917	n = 831	n = 708	n = 564	
Very satisfied		350 (42)	340 (48)	267 (47)	< 0.0001^$^
Satisfied			210 (30)	170 (30)	
Ambivalent		301 (36)	100 (14)	77 (14)	
Dissatisfied			46 (6)	38 (7)	
Very dissatisfied		122 (15)	12 (2)	12 (2)	
* Lost to follow up*		46 (6) 12 (1) *86*	*209*	*353*	

IQR: interquartile range

^*^ p-values for pre-operative vs. each individual postoperative time point

^$^ p-values for six-week vs. each individual postoperative time point

^1^ 59 lost to follow up; ^2^ 179 lost to follow up; ^3^ 353 lost to follow up

^a^ Fisher’s exact test, ^b^ Mann-Whitney U test; ^c^ Chi-squared test

At 6 weeks postoperatively, anxiety/depression had improved to 749 (85%) with no or minimal anxiety/depression, 103 (12%) with moderate symptoms, and 31 (3%) with severe or extreme anxiety/depression (p < 0.0001 compared to preoperatively).

At 6 weeks postoperatively, 433 (49%) patients reported no or slight pain, 358 (41%) had moderate pain, and 94 (10%) reported severe or extreme pain. At six months, 529 (71%) had no or slight pain, 166 (22%) moderate and 52 (7%) had severe or extreme pain. At one year, 434 (75% of 578 respondents) had absent or minimal pain, 105 (18%) had moderate pain symptoms, and 39 (7%) had severe or extreme pain. Both patients who continued using opioids reported severe or extreme pain at one-year follow-up. These incidences are significantly improved compared to preoperative baseline (p < 0.0001).

At six weeks 651 (78%) patients were satisfied, 122 (15%) were ambivalent and 58 (7%) were dissatisfied with their overall experience and surgery (86 lost to follow up for satisfaction questionnaire at six weeks).

PROMs showed an upwards trend across all questionnaires and domains within the questionnaires, consistent with improving function. The Oxford Knee Score showed a significant median postoperative improvement of 11 points at six weeks, and 19 points at one year. (Table 3) Improvements were made at all time points across all PROMs (p < 0.0001).

### Hip arthroplasty group

527 (36%) patients received a hip arthroplasty, who were predominantly female (n = 333; 63%), with a median age of 73 (IQR 66–81) and median BMI of 30 kg/m^2^ (28–36). 187 (35%) patients reported using opioid based medication prior to operation. (Table 4)

**Table 4:** Characteristics for hip arthroplasty patients

	Hips (n = 527)
**Age** in years, median (IQR)	73 (66–81)
**Gender**, n (%)	
Male	194 (37)
Female	333 (63)
**Weight** in kg, median (IQR)	83.0 (70.6–97.2)
**BMI** in kg/m^2^, median (IQR)	30 (26.2–34.9)
**Type of surgery**, n (%)	
Primary	519 (98.5)
Revision	8 (1.5)
**Operative side**, n (%)	
Left	219 (42)
Right	308 (58)
**Pre-operative opiate use**, n (%)	
Yes	187 (35)
No	340 (65)

Abbreviations: IQR: interquartile range

Of the 527 patients who consented to participate in the questionnaire follow-up, 59 (11.2%) were lost to follow-up at six weeks (a slightly higher number of 60 (11.4%) for the satisfaction component). 180 (34.2%) were lost to follow up at six months, and 256 (48.6%) did not respond to the 1 year questionnaire.

At baseline, 344 (66%) patients had no or slight anxiety or depression, moderate anxiety or depression was present in 106 (20%) patients, and 57 (10%) had severe or extreme anxiety or depression. This had improved by six weeks postoperative to 421 (88%) with no or minimal anxiety or depression as part of the EQ-5D-5 L PROM, and 47(10%) with moderate symptoms. 9 (2%) patients had severe and zero had extreme depression/anxiety.

Zero patients in the hip group used opioid based medication after six weeks, this continued out to a year. (Table 5)

**Table 5:** Pre- and postoperative opiate use and patient Reported Outcome Measures for (PROMs) for hip arthroplasty

	Hips (n = 527)	p-value
**Opiate use**, n (%)^a^		
pre-operative	187 (35)	-
6 weeks postoperative^1^	0	< 0.0001^*^
6 months postoperative^2^	0	< 0.0001^*^
1 year postoperative^3^	0	< 0.0001^*^
**Oxford Hip Score total**, median (IQR)^b^		
pre-operative	12 (8–19)	-
6 weeks postoperative^1^	31 (24–37)	< 0.0001^*^
6 months postoperative^2^	38 (31–44)	< 0.0001^*^
1 year postoperative^3^	44 (36–47)	< 0.0001^*^
**EQ-5L-5D Health Questionnaire total**,		
median (IQR)^b^	65 (50–75)	-
pre-operative	80 (70–90)	< 0.0001^*^
6 weeks postoperative^1^	80 (70–90)	< 0.0001^*^
6 months postoperative^2^	85 (75–90)	< 0.0001^*^
1 year postoperative^3^		

At six weeks, 318 (60%) patients had no or slight pain, 134 (25%) had moderate pain, and 26 (5%) reported severe pain. At six months, 297 (85%) had no or slight pain, 39 (11%) moderate and 11 (4%) had severe or extreme pain. At one year, 252 (93% of 271 respondents) had absent or minimal pain, 17 (6%) had moderate pain symptoms, and 2 (1%) had severe or extreme pain. At all postoperative follow-up points, comparison to preoperative incidences were significantly improved (p < 0.0001).

At 6 weeks postoperative 412 (78%) patients were satisfied, 22 (4%) were ambivalent, 27 (6%) were dissatisfied 66 (13%) were lost to follow-up. This remained consistent at six months and one year. This represented a significant change in incidence (p < 0.0001).

The Oxford Hip Score showed a median postoperative improvement of 19 points at six weeks, and 31 points at a year. (Table 6) Statistically significant improvements were made at all time points across all PROMs (p < 0.0001).

**Table 6:** Pre- and postoperative patient Reported Outcome Measures (PROMs) for hip arthroplasty

	Pre-operatively	6 weeks	6 months	1 year	p-value
**EQ-5L-5D pain/discomfort score**, n (%)^c^	n = 527	n = 477	n = 347	n = 271	
No pain	3 (1)	111 (23)	181 (52)	200 (73)	< 0.0001^*^
Slight pain	32 (6)		116 (33)	52 (19)	
Moderate pain	152 (29)	207 (43)	39 (11)	17 (6)	
Severe pain	187 (235)		10 (3)	1 (1)	
Extreme pain	153 (29)	134 (28)	1 (1)	1 (1)	
* Lost to follow up*	-	24 (5) 1 (1) *50*	*180*	*256*	
**EQ-5L-5D anxiety/depression score**, n (%)^c^	n = 507	n = 477	n = 345	n = 269	
Not anxious/depressed	199 (38)	332 (70)	257 (74)	210 (78)	< 0.0001^*^
Slightly	145(28)	89 (18)	62 (18)	43 (16)	
anxious/depressed	106 (20)	47 (10)	20 (6)	13 (5)	
Moderately	34 (6)	9 (2)	6 (2)	3 (1)	
anxious/depressed	23 (4)	0	0	0	
Severely	*20* ^*^*^	*50*	*182*	*258*	
anxious/depressed					
Extremely					
anxious/depressed					
* Lost to follow up*					
**Likert patient satisfaction**, n (%)^c^	n = 527	n = 461	n = 317	n = 240	
Very satisfied		291 (63)	203 (64)	157 (65)	< 0.0001^$^
Satisfied			80 (25)	60 (25)	
Ambivalent		121 (26)	17 (5)	11 (5)	
Dissatisfied		22 (5)	5 (2)	3 (1)	
Very dissatisfied		8 (2)	12 (4)	9 (4)	
* Lost to follow up*		19 (4) *66*	*210*	*287*	

IQR: interquartile range

^*^ p-values for pre-operative vs. each individual postoperative time point

^^^ declined to answer

^$^ p-values for six-week vs. each individual postoperative time point

^1^ 59 lost to follow up; ^2^ 180 lost to follow up; ^3^ 256 lost to follow up

^a^ Fisher’s exact test, ^b^ Mann-Whitney U test; ^c^ Chi-squared test

## Discussion

This study demonstrates that a patient education protocol with emphasis on patient expectation management coupled with a multi-disciplinary approach to pain management can result in a long-term opioid free recovery. This is supported on a national level in Australia by multiple programs designed to restrict and monitor opioid prescribing. Similar programs in other countries have also shown success in previous studies. [21, 22]

The subject of opioid use for pain management is ‘one of intense international interest’, according to Morgan et al. [23] Dependence can develop following the use of prescription opioids after surgery. [24] Health systems internationally have flagged this as an area of concern and strategies aimed at minimising postoperative opioid prescriptions are increasingly suggested. [25] That said, opioids are a cornerstone of postoperative pain management and inadequate analgesia can result in delayed mobilisation and recovery. [26] The results of the current prospective study, however revealed no decrease in PROM outcomes during an opioid free postoperative phase, showing that joint arthroplasty surgery can be managed with simple analgesia postoperatively without compromising quality of recovery. Pain scores are also comparable with previously published studies, if not lower. [27, 28] Pain scores at 6 weeks postoperatively for TKA were described in previous studies as moderate (approximately 5 on a 11 point Likert scale), and decreasing for the following 12 months postoperatively. Another study found at 12 months postoperatively for TKA that 40% of patients had moderate to severe pain. Chronic pain and dissatisfaction have been reported as being as high as 10–34% of patients at 12 months after TKA. [29] Our study describes perhaps less pain, with a majority reporting only slight pain at 6 weeks and moderate pain being the second most common response with a similar pattern of pain improvement over the 12 months of follow-up.

Patient satisfaction rates at our institution are consistent or better than reported incidences from other tertiary centres, [30] describing dissatisfaction rates of up to 20% under a classic opioid prescribing regime. [31] This may, however, not be related to the opioids specifically, but likely also to the hands-on care, meticulous preoperative preparation, and extensive follow up.

Twenty-five (5%) hip patients reported severe or extreme postoperative pain, and 94 (10%) knee patients at six weeks postoperatively. The limitation to this result is that data collection did not specify the location of and frequently patients were reporting pain in the contralateral joint, likely due to the same underlying pathology. Interestingly, these patients did not report dissatisfaction with the surgery, nor did they use postoperative opioids, making it plausible their pain was indeed not related to the operated joint.

The two patients who remained on opioids postoperatively both recorded preoperative opioid use and severe anxiety and/or depression at all time points. PROM improvements were consistent with average results from the group. One patient reported low patient satisfaction, and both had continuing severe pain despite the prescription of opioid medication.

We compare our incidence of 2/1444 patients who remained on long term opioid medications with previously published incidences of Australian hip and knee arthroplasty patients who followed a traditional regime, i.e. not opioid sparse. It has been described in a similar population as our own as 10% at 6 months postoperatively following TKR, and 4% at 6 months postoperatively following THR. [32]

Despite the common use of prescription opioids for chronic non-cancer pain, there remains no strong scientific evidence to support this routine practice. [33] This study illustrates that recovery from arthroplasty surgery can be achieved with good patient satisfaction and high-quality PROM outcomes, while remaining opioid sparse. Furthermore, opioid use is associated with an increase in long-term utilisation of health care services, as well as inflicting a significant economic burden. [34]

PROM surveys and long-term opioid based data collection will be integrated shortly into the national Australian Orthopaedic Association joint registry database. [35] This promises to provide an interesting look into opioid prescription and recovery on a large scale in the future. It is likely that the presence of an Acute Pain Service, as well as the use of novel regional anaesthesia assisted in this outcome.

## Limitations

Despite the favourable outcomes described in this study, some limitations do need to be addressed. There is unfortunately no historical data collection at our centre prior to implementation of this protocol, and hence we cannot definitively conclude that a change has been actuated. We have compared to published data in other centres, with similar patient populations and more classic opioid regimes to illustrate the difference. These prescription rates are self-reported by patients, which is also a limitation.

We acknowledge that there may in fact be a cultural component to the result of a low opioid prescription rate, where the team approach is to ‘just say no’ to continuing postoperative opioids. We would argue that if that is a factor in our positive outcomes, that it is not necessarily a weakness. Our high patient satisfaction rate and good PROM results despite our low opioid prescription rates show that it is attainable.

It is also possible that the patients who were lost to follow-up were taking opioids. It is unfortunately not possible for us to determine if this is the case. The pattern of lost to follow-up is that the percentage attrition is greater the further away from index surgery, which is not consistent with attrition due to opioid prescribing rates in the acute postoperative phase continuing from surgery. Non-responders had comparable baseline characteristics to responders, i.e. were not more likely to be using opioids preoperatively. The attrition rate, for example 6.4% at 6 weeks for TKA, is also lower than most opioid prescribing rates described in previously published studies. We hope that in the near future with the implementation in Australia of Script Check, that it becomes easier to determine patient opioid use across all health providers.

## Conclusion

In conclusion, the results of this multi-centre prospective study show no decrease in PROM outcomes or patient satisfaction during an opioid free postoperative phase for the vast majority of patients. This illustrates that joint arthroplasty surgery can be managed with non-opiod analgesia postoperatively between six weeks and one year, without compromising pain scores or quality of recovery. This topic should be considered for future investigation by means of a randomized controlled trial.

## Electronic supplementary material

Below is the link to the electronic supplementary material.

Supplementary Material 1

## Data Availability

The datasets generated and/or analysed during the current study are not publicly available as this was not approved by the Ethics Committee but are available in a deidentified format from the corresponding author on reasonable request.

## List of abbreviations

PROMs: Patient reported outcome measures
LIA: Local infiltration analgesia
PENG: Pericapsular nerve group block
BMI: Body mass index
OKS: Oxford Knee Score
OHS: Oxford Hip Score
