# Supplementary material for: A multi-disciplinary program for opioid sparse arthroplasty results in reduced long-term opioid consumption: a four year prospective study
Source: BMC Anesthesiol. 2023 Mar 29;23:97. doi: 10.1186/s12871-023-02062-8 (PMC10050824; doi:10.1186/s12871-023-02062-8)
Supplement: Supplementary file 1 — Supplementary Material 1 [file 12871_2023_2062_MOESM1_ESM.docx]

Addendum 1.

Arthroplasty Patient Care Protocol

PREOPERATIVE

Patient is booked for surgery and stratified according to urgency

Patient is referred to Anesthesiology for assessment, and physiotherapy for prehabilitation

Primary care physician is advised of upcoming surgery, and to taper dose of any opioid medications

Regular use of simple analgesia is advised

Patient attends a mandatory patient education session 4 weeks prior to surgery

INTRAOPERATIVE

Premedication of oral paracetamol

Anesthesia technique is suggested to be spinal anesthesia, and use of regional blocks:

PENG (Pericapsular Nerve Group) block for hip replacement is recommended

Adductor canal +/- IPACK (Infiltration between the Popliteal Artery and Condyles of the Knee) block for knee replacement

No intrathecal morphine, or low dose (100mcg) only

LIA (Local Infiltration Analgesia) 100mLs of 0.1% ropivacaine

POSTOPERATIVE

Early mobilisation with physiotherapy, same day as surgery

Regular simple analgesia

Opioid based medication allowed on a PRN only basis, if indicated as inpatient

Cessation of any slow-release opioid medication

Daily or twice daily physiotherapy

Daily ward rounds with reinforcement that the patient should be aiming to be opioid free

Daily review by Acute Pain Service with reinforcement that the patient should be aiming to be opioid free

DISCHARGE

Advised to continue regular simple analgesia if needed

Preference for no opioid on discharge. If opioid discharge script given- provided for a maximum of ten tablets, no repeats

Follow-up appointments with arthroplasty team at six weeks and three months

Written communication to primary care physician, reinforcing expectation of opioid-free postoperative trajectory

Follow-up with research assistant at six weeks, six months and one year to assess PROMs, patient satisfaction and opioid use.
